# Severe Sydenham’s chorea (*chorea paralytica*) successfully treated with plasmapheresis

**DOI:** 10.1186/s40734-014-0012-1

**Published:** 2015-01-21

**Authors:** Marcelo Miranda, Ruth H Walker, David Saez, Victoria Renner

**Affiliations:** Department of Neurology, Clinica Las Condes, Santiago, Chile; Department of Neurology (127), James J. Peters Veterans Affairs Medical Center, 130 W. Kingsbridge Road, Bronx, NY 10468 USA; Mount Sinai School of Medicine, New York, USA; Department of Neurology, Hospital Barros Luco, Santiago, Chile

**Keywords:** Sydenham’s chorea, Plasmapheresis, Chorea paralytica

## Abstract

Sydenham’s chorea is often regarded as a relatively benign and self-limiting condition. Treatment is typically symptomatic, although occasionally immunomodulatory therapies are required in severe forms. Here we report a girl who was affected with the severe variant, *chorea paralytica,* who responded dramatically and rapidly to plasmapheresis, having failed other therapies.

**Electronic supplementary material:**

The online version of this article (doi:10.1186/s40734-014-0012-1) contains supplementary material, which is available to authorized users.

## Background

Sydenham’s chorea (SC) is the neurological manifestation of rheumatic fever, a complication of group A beta-hemolytic streptococcal infection. Although the incidence of SC has declined markedly, particularly in developed countries, it remains the most prevalent cause of acute chorea in children worldwide [1]. SC accounts for almost all cases of acquired chorea in childhood in the United States [2].

SC is considered to be a humorally-mediated autoimmune disorder and, in addition to chorea, is characterized by decreased muscle tone, motor impersistence, and psychiatric and behavioral symptoms, particularly obsessive-compulsive symptoms and hyperactivity [1,3]. The usual age at onset of SC is around 9 years old with a female predominance [1].

This condition is usually managed with anti-choreic drugs, such as valproic acid and neuroleptics, in addition to benzathine penicillin prophylaxis to prevent recurrence of streptococcal infection [1].

Despite treatment with valproic acid and neuroleptics, 5% of patients with SC fail to respond [1,4]. We report a patient with a severe form of SC, the so-called *chorea paralytica* or *chorea mollis* [1], who responded dramatically and rapidly to plasmapheresis after failure of conventional therapy and intravenous corticosteroids.

## Case presentation

Two weeks following a throat infection, a previously healthy 16-year-old girl acutely developed generalized involuntary movements. Within a few days, she became bedridden, incapable of standing or walking without assistance, and requiring a naso-gastric tube for feeding. On initial examination, the most relevant clinical findings were lack of spontaneous speech, generalized chorea, and a severe decrease in muscle tone, to the extent that she was unable even to sit on the bed (Additional file 1), corresponding to *chorea paralytica*. Strength and deep tendon reflexes were normal. No other abnormal signs were observed. Antistreptolysin O (ASO) titer was elevated (300 U, normal value <200), as was anti-DNase B titer (300 units/mL, normal <170 units/mL). There were no abnormalities of serum glucose, antinuclear antibodies, antiphospholipid antibodies, thyroid function tests or syphilis serology. Cerebrospinal fluid and brain MRI were normal. Echocardiogram revealed no signs of carditis. Anti-basal ganglia antibody testing was not available. There was no family history of neurologic disease or use of any drug which might induce a movement disorder.

The diagnosis of SC was made, and she was treated with valproic acid (maximum dose 1.5 g/day) and risperidone (maximum dose 3 mg/day) without a reduction in chorea. Methylprednisolone 1 g i.v daily for 3 days followed by prednisone 1 mg/kg p.o. produced no change in her neurologic status. Five rounds of plasmapheresis were performed with a rapid improvement in hypotonia, starting 3 days after the last treatment (Additional file 1). Marked improvement persists at 1 month of follow-up (Additional file 1). She remains on benzathine penicillin prophylaxis since hospital discharge.

## Conclusions

SC is often considered a benign, self-limiting condition that usually spontaneously remits after 6–7 months. However, in contradiction to this perception, Cardoso et al. found that after 2 years 50% of SC subjects continue to have disabling persistent chorea, despite ongoing treatment [4]. SC can be very disabling even at presentation, as in our case.

There are very few reports regarding the use of plasmapheresis in SC. In an unblinded study of 18 patients treated with plasmapheresis, Garvey et al. reported that 8 cases had a marked response [5].

Our report supports the early use of plasmapheresis in severe cases of SC (*chorea paralytica*) which may be less responsive to conventional symptomatic and immunomodulatory therapy, such as corticosteroids. Although their biological role has not been unequivocally demonstrated, anti-basal ganglia antibodies (ABGA) have been found in 100% of patients with acute SC [6]. Although plasmapheresis is regarded as an experimental therapy in SC [1,7], the presence of these ABGA provides a rationale for its use [5,8,9]. Historical reports demonstrate that severely affected children improve more slowly than those with milder symptoms. In the Garvey study of 18 moderate and severe cases [5], as in the present case, there was a rapid and dramatic response, [5], occurring during one month of therapy, or in a few days as seen here.

In cases of *chorea paralytica* it is unreasonable to wait months for a response after conventional therapy, and a more aggressive approach is justified. However, at the present time, according to evidence-based guidelines, there is insufficient evidence to support or refute the use of plasmapheresis in Sydenham chorea (Class III evidence, Level U) [7].

## Consent

Written informed consent was obtained from the patient’s parents for publication of this case report and any accompanying images. A copy of the written consent is available for review by the Editor-in-Chief of this journal.

## Additional file

Additional file 1:
**Segment 1 shows the patient before treatment with severe hypotonia and moderate-severe generalized chorea.** In segment 2, 3 days after completing plasmapheresis, there is a marked reduction in hypotonia, mild-moderate chorea, occasional myoclonus, and motor impersistence. At 1 month after treatment she has only mild chorea (segment 3).

## Abbreviations

ABGA: Anti-basal ganglia antibodies
ASO: Antistreptolysin O
SC: Sydenham’s chorea
